# Job vacancies at the European Centre for Disease Prevention and Control (ECDC)

**DOI:** 10.2807/1560-7917.ES.2023.28.42.2310196

**Published:** 2023-10-19

**Authors:** Eurosurveillance editorial team

**Affiliations:** 1 European Centre for Disease Prevention and Control (ECDC), Stockholm, Sweden

The European Centre for Disease Prevention and Control (ECDC) invites applications for the following positions:

 

**Scientific Officer Prevention and Behaviour Change**
The application deadline expires on 15 Nov 2023.

  

**Scientific Officer Vaccine-Preventable Diseases**
The application deadline expires on 17 Nov 2023.

For more information, please visit the ECDC website: https://erecruitment.ecdc.europa.eu/?page=advertisement

